# Prevalence and Health Correlates of Work-Life Conflict among Blue- and White-Collar Workers from Different Economic Sectors

**DOI:** 10.3389/fpubh.2014.00221

**Published:** 2014-11-11

**Authors:** Oliver Hämmig

**Affiliations:** ^1^Epidemiology, Biostatistics and Prevention Institute, University of Zurich, Zurich, Switzerland

**Keywords:** blue-collar workers, white-collar workers, work-life conflict, self-rated health, back pain, sleep disorders, Switzerland

## Abstract

The research on work-life conflict (WLC) is largely neglected in occupational medicine and public health and typically limited to white-collar workers and public servants. This study therefore aims to explore possible differences in the prevalence of WLC and its association with health outcomes between white- and blue-collar workers from different work environments in Switzerland. Cross-sectional survey data collected in 2007 in the service sector and in 2010 in the industrial sector were used for statistical analyses. A subsample of university graduates employed by large service companies (*N* = 1,170) from the first survey’s population was taken and compared with a subsample of low or unskilled industrial and construction workers with no or only compulsory education (*N* = 489) from the second survey’s population. The results show almost consistently, and particularly in women, a lower prevalence of time- and strain-based forms and both causal directions of WLC in blue-collar workers. However, associations between different WLC measures and general, physical and mental health outcomes were found to be equally strong or even stronger among blue-collar workers compared to white-collar workers. Low or unskilled industrial and construction workers are less frequently affected by higher degrees of WLC but are then at no lower risk of suffering poor self-rated health or severe backaches and sleep disorders than university graduates working in the service sector with comparable exposure to WLC. In conclusion, it can be stated that WLC turned out to be much less prevalent but equally or even more detrimental to health in blue-collar workers, who therefore need to be considered in future studies.

## Introduction

Inter-role conflict and negative spillover between paid work and family or personal life, also referred to as work-home interference, work-family conflict, or work-life conflict (WLC) as the more inclusive term, is studied predominantly in psychology and particularly in occupational health psychology (1–3). This construct is conceptualized as multidimensional and particularly bidirectional, and is classified by three forms (time-based, strain-based, and behavior-based) and two directions (from work-to-family/life and from family/life-to-work) of conflict or spillover (4–8). It was originally introduced by Greenhaus and Beutell (9) already 30 years ago. However, until recently the phenomenon and concept of WLC was largely ignored in occupational medicine and traditional occupational health research as well as in public health research and literature, but has now been recognized as an important psychosocial work factor and public health issue (10). In particular and recent years, WLC has been found to be a strong work-related stressor and a major risk factor for specific mental and musculoskeletal disorders (11–15), which are among the most prevalent and relevant work-related health problems.

The extensive research on the causes, consequences, and measurement of work-family conflict (1, 16–18) or WLC, is limited in many respects and has been criticized for various methodological and theoretical deficiencies, shortcomings, and gaps (1, 3, 8, 19–22). In previous studies on the subject matter, we have addressed some of these limitations with regard to sample heterogeneity and restriction, and study design (11–15, 23–25). Another major limitation or criticism that is made and that we have not yet addressed is a widespread middle-class bias and, thus, a limited scope of the research (3, 19). Most studies have used middle-class samples and were conducted with high-status professionals and well-educated employees of large organizations or public administrations [see *inter alia* (1, 8)]. Poorly qualified employees or unskilled manual workers and members of lower classes or ethnic minorities have, with very few exceptions (26–30), been widely neglected and hardly investigated at all in this regard. Accordingly, very little is known so far about the work-life experiences of blue-collar workers, and although associations between WLC and health impairments are well and broadly supported by the evidence, this has been shown almost exclusively for middle-class employees in white-collar jobs, hence strongly limiting the possibility of generalizing the findings.

The present study aimed to uncover this blind spot of WLC research and to fill the research gap and lack of evidence by examining both white- and blue-collar workers from two fundamentally different work environments, or rather economic sectors (industrial, service), and comparing them with regard to the prevalence and health outcomes of WLC.

### Theoretical considerations

Research on WLC is dominated by the role strain hypothesis, which postulates that role strain – defined as the difficulty in fulfilling role expectations – results from participation in occupying multiple roles and particularly from the many work demands and family obligations placed on individuals, which compete with each other for limited amounts of time, energy, and psychological resources (5). From this perspective and given the different nature of blue-collar and white-collar jobs, it seems obvious and can be expected that unskilled or poorly skilled industrial workers (“blue-collar workers”) are less likely to experience WLC or negative spillover from work to personal life (and vice versa) than highly qualified service workers (“white-collar workers”). There is evidence to support this assumption: it has been shown previously and repeatedly among middle-class and white-collar workers that higher ranking and better educated employees experience such WLC more frequently than employees in lower job positions and/or with lower levels of education (11, 12).

As is well-known, many blue-collar and lower-income workers have physically demanding jobs and typically work under adverse, strenuous, and precarious conditions (e.g., monotonous work, highly repetitive work, lifting and carrying heavy loads, poor posture, and night or shift work). In contrast, white-collar workers generally have more mentally and/or emotionally demanding jobs and not unusually work under difficult and stressful psychosocial conditions (e.g., irregular or long hours, high time pressure, frequent interruptions, growing workload, and job insecurity). Such demanding psychosocial working conditions may be more likely to spill over to private life and interfere with family roles, household duties, and leisure activities than adverse physical working conditions. It is presumably easier to reconcile private and family life activities with high physical demands and strenuous working conditions than with high psychosocial demands and stressful working conditions.

On the other hand, it is evident and undisputed that unskilled and poorly qualified employees mostly have fewer resources at home and at work and less control over their living and working conditions than highly qualified and higher earning employees. In particular, they experience lower levels of supervisor support, flexibility, or autonomy at work and work time control (26). And social support (at work) as well as job control or job autonomy have been repeatedly shown to offer at least partial protection from job stress and role strain at work (WLC) and provide a buffer against the negative health consequences and organizational outcomes of job stressors such as WLC [see *inter alia* (11, 31)]. Consequently, once exposed to stressful interference and negative spillover between the two life domains, low-income and blue-collar workers, although possibly less frequently confronted with such conflict, are expected to suffer more strongly from it and to be more vulnerable to negative spillover due to their lack of resources, and particularly of job control.

**Figure 1:**
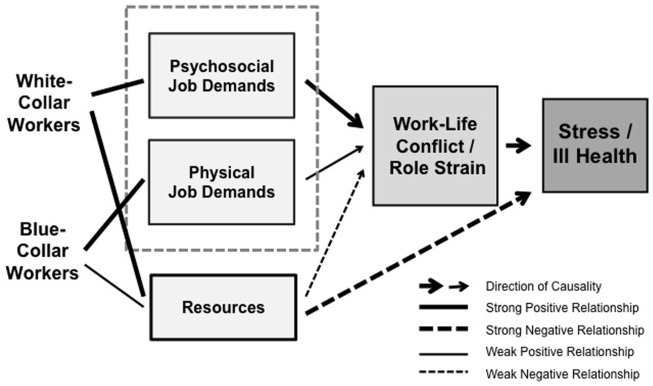
**Hypothesized (causal) relationships and pathways underlying the study and illustrated for the two different study populations**.

In the present comparative study, these assumptions of a lower prevalence but higher health impact of WLC in blue-collar compared to white-collar workers will be studied by contrasting two independent subsamples of employees from two large-scale organizational surveys in Switzerland carried out at a distance of 3 years, and by using identical measures for general, physical, and mental health outcomes and different aspects or dimensions of WLC. The path model in Figure 1 illustrates the hypothesized causal relationships underlying the study and leading to the postulated differences between blue- and white-collar workers with regard to prevalence of WLC and its association with health.

## Materials and Methods

### Data and study sample

The present study is based on cross-sectional data from two employee surveys on the broad topic of work and health with extraordinary large and heterogeneous samples representing all kinds of educational and hierarchy levels. The postal and online surveys were conducted in the tertiary or service sector (survey I) in 2007 and in the secondary or industry sector (survey II) in 2010. Data were collected by full and sample surveys among the workforces of eight medium-sized and large companies from various industries (construction, machine, chemistry and biotechnology, watch and knife production, insurance, banking, transportation, and healthcare). For both surveys, a two-stage selection procedure was implemented with self-selection by the participating companies and a complete (seven companies) or simple random (one company) sampling of the employees to be interviewed. The only criteria for inclusion in the study were the general willingness of the companies to participate and their representation of different industries. However, subsamples were representative of the companies’ workforces but not of the corresponding industry or economic sector.

The return or response rates among the companies were between 35 and 68%. Overall participation rates were 56.0% (survey I), 49.3% (survey II), and 54.2% (survey I & II). In the employee survey of 2007, a total of 6,091 employees were interviewed and returned their questionnaires online or by mail, and 5,930 of them gave information about their education (with 161 missing values). In the employee survey of 2010, a total of 2,014 interviews (written questionnaires) were completed, and 1,960 of them included information about education (with 54 missing values). Such information is important, since education has been used as a key criterion for the selection of the study samples (blue- and white-collar workers) out of these two survey populations (see Table 1).

**Table 1:** **Characteristics of study samples (white- and blue-collar workers) in comparison with entire survey populations and a nationally representative standard population**.

		Standard population	Survey population I	Study sample I	Survey population II	Study sample II
		Swiss Household Panel 2007 (*N* = 3,885)	Employee survey 2007 in the service sector (*N* = 5,930)	Subsample of university graduates (*N* = 1,170)	Employee survey 2010 in the industry sector (*N* = 1,960)	Subsample of low or unskilled workers (*N* = 489)
		**%^a^**	**%**	**%**	**%**	**%**
**Sex**	Men	49.0	57.1	69.4	82.2	74.0
	Women	51.0	42.9	30.6	17.8	26.0
**Age**	(15)–30 years	28.4	24.9	15.6	20.2	24.8
	31–40 years	23.0	31.9	46.7	22.2	13.2
	41–50 years	26.0	27.4	28.3	31.3	31.6
	51 years and older	22.5	15.8	9.4	26.2	30.4
**Education** (highest level achieved)	**No compulsory or vocational education**	19.1	5.8	−	**24.9**	**100.0**
	Basic vocational education	39.2	37.1	−	46.8	−
	Higher vocational education	25.8	37.3	−	19.4	−
	**University degree**	15.9	**19.7**	**100.0**	8.8	−
**Nationality**	Swiss (incl. dual citizenship)	78.8	87.6	68.4	86.7	82.2
	Other nationality	21.2	12.4	31.6	13.3	17.8
**Job status** (occupational position)	Management position (directorate)	4.7	5.2	6.5	0.7	0.0
	Supervisory pos. (executive staff)	24.1	34.7	53.1	28.6	9.1
	Production position (regular staff)	71.2	60.1	40.3	70.7	90.9
**Employment level**	Part-time ( <50%)	17.4	5.4	1.5	0.9	0.6
	Part-time (50 to <100%)	24.2	21.5	15.0	7.5	11.5
	Full-time (100%)	58.4	73.1	83.5	91.6	87.9

*^a^Based on a representative and weighted random sample of the resident and working population in Switzerland and restricted to a subsample of employees of private and public organizations in the working age range between 15 and 65*.

These two survey populations differ significantly from each other and from a comparable standard population, a nationally representative sample of the general working or rather employed population in Switzerland, with regard to education and several other sociodemographic characteristics (see Table 1). In survey population I (from the service sector), academics and other highly educated employees are strongly overrepresented (57.0%) compared to the standard population (41.7%), as are executive employees (39.9 vs. 28.8%) and Swiss citizens (87.6 vs. 78.8%). In survey population II (from the industrial sector), employees with no or only a basic vocational education (71.7 vs. 58.3%), men (82.2 vs. 49.0%), and hence full-time employees (91.6 vs. 58.4%) are strongly overrepresented. Although both survey populations include blue-collar workers such as unskilled baggage porters, cleaners, or assembly line and construction workers as well as white-collar employees such as private or investment bankers, risk managers, engineers, key account managers, salesmen, or physicians, survey population I is clearly dominated by high-income and white-collar workers, whereas low-income and blue-collar workers are in the majority in survey population II.

In order to compare extreme groups with regard to education (academics vs. unskilled workers) and working conditions (mainly office work vs. manual work) and to have two independent samples consisting largely of white- and blue-collar workers, respectively as far as possible, the present study was restricted to a subsample of each of the two survey populations. Inclusion criteria for being selected were the level of education (highest vs. lowest) combined with employment in either the secondary or the tertiary sector of the economy (industry vs. service). The subsample of university graduates from the first survey’s population was taken and compared with a subsample of low or unskilled industrial or construction workers with no or only compulsory education from the second survey’s population (see Table 1). These two subsamples again differ from each other and from the corresponding survey populations with regard to several sociodemographic characteristics (see Table 1).

### Measures

#### Work-life conflict

Work-life conflict was measured almost identically in the two employee surveys with a multi-item scale, or rather four multi-item subscales and a commonly used 4-point single-item general indicator of the degree of reconcilability of work hours with family and private obligations. The 5-point Likert scaled items of the multi-item subscales were taken from the well-established and validated 18-item work-family conflict scale of Carlson et al. (17), translated into German and slightly reformulated by replacing or complementing the term “family” with “private life” or other expressions comprising the whole non-work domain. This was done to avoid an unwanted exclusion of singles, childless employees, or older workers with grown-up children (no longer living in the same household) from study participation and, hence, to preclude an inappropriate restriction of the study population only to those working men and women with children (and spouse) living at home. The multi-item measures of both surveys covered the same four out of six recognized dimensions of the construct, namely the two most frequently used forms of each causal direction. These four dimensions are the time-based work-to-life conflict (e.g., “I regularly miss private events or family activities because of my work”), the strain-based work-to-life conflict (e.g., “When I come home from work, I often feel too drained to take part in family or private activities”), the time-based life-to-work conflict (e.g., “My family and personal obligations often keep me from participating in work events, which are important for my career”), and the strain-based life-to-work conflict (e.g., “Family tensions or personal worries often lower my performance at work”). In line with many other studies in this field [see *inter alia* (13, 18, 32)], the behavioral-based form of both directions was not included in the questionnaire and therefore not considered in this study.

Survey II used all 12 adapted and translated items from the multi-item scale of Carlson et al. (17) (reduced only by the six items of excluded behavioral-based form in both directions), whereas survey I included only 11 items from the original scale and in identical terms and made them available as measures or indicators of the four covered dimensions. More precisely, the time-based life-to-work conflict in survey I was measured by only two instead of three items. With this single exception, three identical items were used for each of the measured dimensions or subscales in both surveys. The 5-point items with response categories and values ranging from “Completely disagree” (score 0) to “Completely agree” (4) were added up and, for further and comparative analyses, recoded into three different levels of conflict (no/low, moderate, and high) according to the total score.

The reliability of the adapted and shortened WLC subscales based on translated and modified items has been assessed by testing the internal consistency and calculating Cronbach’s alpha coefficients. Reliability analyses for both study samples with the exception of the above mentioned two-item WLC subscale (time-based life-to-work conflict) in the white-collar workers’ sample (study sample I) resulted in fairly good up to very good Cronbach’s alpha coefficients for all WLC subscales. The alpha coefficients (α) were similarly high in the two study samples for each of the WLC dimensions, the time-based work-to-life conflict (α = 0.81 in the white-collar workers’ sample and α = 0.85 in the blue-collar workers’ sample), the strain-based work-to-life conflict (α = 0.89 and 0.91), the time-based life-to-work conflict (α = 0.53 and 0.79), and the strain-based life-to-work conflict (α = 0.84 and 0.85).

#### Health outcomes

The general, physical, and mental health outcomes used in this study are self-rated health (SRH), backache, and sleep disorders and were measured identically in the two surveys. SRH is a commonly used single-item measure of general health status in epidemiology and social science. According to Idler and Benyamini (33) and their most often cited systematic review of 27 community studies, global self-ratings of health represent a source of very valuable and irreplaceable data on an individual’s health status. They consistently found and concluded that global self-ratings of health are valid, strong, and independent predictors of overall mortality, measuring something more than objective medical ratings and adding something more to the prediction of mortality (33). SRH was measured by a 5-point Likert scaled item with response categories ranging from “Very well” (1) to “Very poor” (5). Backache and sleep disorders were measured by a question taken from the Swiss Health Survey on having suffered pain or medical complaints such as back or lower back pain as well as difficulty in sleeping or insomnia in the last 4 weeks, with three possible answers each (Yes, severely/Yes, a little/No, not at all).

### Analyses

First sex-specific relative frequencies of diverse levels and scores or value ranges of the single-item general indicator and the four multiple-item subscales of WLC were calculated for both survey populations and both study samples. Data sets of the two surveys were pooled, which allowed us to test for statistically significant differences between the two survey populations and study samples. Stratified logistic regression analyses were then performed for both study samples, and sex- and age-adjusted odds ratios were calculated as measures of association and indicators or rather proxies for relative risks. For this purpose and due to the skewed distributions or “only” three response categories, health outcomes were recoded to binary variables. Odds ratios were estimated for each additional level or score of the original from four up to 12-point scaled WLC measures and additionally by using the indicator method and the recoded variables on a three-point scale i.e., by contrasting the more and most exposed groups with the least exposed reference group experiencing no or low WLC or having work hours that are highly compatible with private life activities and obligations. The stratification of the analyses naturally did not allow to test for a statistical significance of the differences between associations of WLC measures and health outcomes in the two study samples.

## Results

Low compatibility of work hours with private life activities and higher levels of time- and strain-based WLC (with the exception of strain-based life-to-work conflict) turned out to be more frequent in the service sector and particularly among white-collar workers than in the industry sector, and especially among blue-collar workers. The statistically significant difference in relative frequency of WLC that could be observed between the two sectors or survey populations (see Table 2) was even more pronounced when extreme groups or subsamples of highly qualified service workers (study sample I) and low-skilled or unskilled industrial and construction workers (study sample II) were compared (see Table 3). This applies in particular to the time-based forms of WLC for which differences were found to be more distinct, and to women who also showed a bigger difference between white- and blue-collar workers in this regard than men (see Table 3). In women, 21% of university graduates employed in the service sector and by large organizations, but only 1% of low-skilled or unskilled industrial and construction workers reported having work hours, which were incompatible with private life activities. In addition, 49% (19%) of white-collar employees but only 14% (3%) of blue-collar workers showed moderate to high levels of time-based work-to-life (life-to-work) conflict. And as many as 64% (10%) of white-collar workers compared to 54% (6%) of blue-collar workers were affected by high strain-based work-to-life (life-to-work) conflict.

**Table 2:** **Prevalence of different work-life conflict measures among male and female employees in the service and industry sectors (survey populations I and II)**.

	Men			Women			Total		
	Survey population I	Survey population II	*p*	Survey population I	Survey population II	*p*	Survey population I	Survey population II	*p*
	(*N* = 3,368)	(*N* = 1,609)		(*N* = 2,526)	(*N* = 349)		(*N* = 5,930)	(*N* = 1,960)	
	%	%		%	%		%	%	
Compatibility of work hours with private life activities			***			***			***
Very good	17.5	33.2		20.8	44.1		18.8	35.2	
Good	65.2	50.3		61.0	49.6		63.4	50.2	
Not very good/ not good at all	17.4	16.5		18.2	6.3		17.8	14.7	
Time-based work-to-life conflict			***			***			***
None or low (0–4)	56.8	60.0		57.9	73.6		57.2	62.4	
Moderate (5–7)	32.0	24.5		29.8	17.2		31.1	23.2	
High (8–12)	11.2	15.5		12.3	9.2		11.7	14.4	
Strain-based work-to-life conflict			***			**			***
None or low (0–4)	46.2	54.8		40.1	48.7		43.5	53.7	
Moderate (5–7)	34.4	29.7		37.1	33.4		35.5	30.4	
High (8–12)	19.4	15.5		22.8	17.9		20.9	15.9	
Time-based life-to-work conflict			***			**			***
None or low (0–2/0–4)	85.4	93.6		88.0	93.7		86.5	93.6	
Moderate (3–5/5–7)	13.8	5.3		10.9	5.2		12.6	5.3	
High (6–8/8–12)	0.8	1.1		1.2	1.2		0.9	1.1	
Strain-based life-to-work conflict			n.s.			n.s.			n.s.
None or low (0–4)	87.8	89.3		91.5	93.4		89.4	90.1	
Moderate (5–7)	10.2	9.0		7.0	6.4		8.8	8.6	
High (8–12)	2.0	1.6		1.6	0.3		1.8	1.4	

Chi-square test: n.s., not significant (*p* > 0.05); *p ≤ 0.05; **p < 0.01; ***p < 0.001.

*Note: The sum of numbers of men and women does not equal the total number of participants in both survey populations due to missing values with respect to sex*.

**Table 3:** **Prevalence of different work-life conflict measures among male and female white- and blue-collar workers from the service and industry sectors (study samples I and II)**.

	Men			Women			Total		
	Study sample I	Study sample II	*p*	Study sample I	Study sample II	*p*	Study sample I	Study sample II	*p*
	(*N* = 808)	(*N* = 361)		(*N* = 357)	(*N* = 127)		(*N* = 1,170)	(*N* = 489)	
	%	%		%	%		%	%	
Compatibility of work hours with private life activities			***			***			***
Very good	16.3	36.2		19.9	49.6		17.4	39.8	
Good	61.0	50.3		58.7	49.6		60.3	50.1	
Not very good/ not good at all	22.7	13.5		21.3	0.8		22.3	10.1	
Time-based work-to-life conflict			***			***			***
None or low (0–4)	51.0	66.5		51.0	85.7		51.0	71.5	
Moderate (5–7)	34.4	20.5		31.2	11.9		33.4	18.3	
High (8–12)	14.6	13.0		17.8	2.4		15.6	10.3	
Strain-based work-to-life conflict			***			*			***
None or low (0–4)	44.1	58.1		35.9	46.4		41.6	55.1	
Moderate (5–7)	34.0	27.8		37.3	36.8		35.0	30.1	
High (8–12)	21.9	14.2		26.8	16.8		23.4	14.8	
Time-based life-to-work conflict			***			***			***
None or low (0–2/0–4)	82.6	90.0		80.7	96.8		82.1	91.8	
Moderate (3–5/5–7)	16.3	8.3		16.4	3.2		16.3	7.0	
High (6–8/8–12)	1.1	1.7		2.8	0.0		1.6	1.2	
Strain-based life-to-work conflict			n.s.			n.s.			n.s.
None or low (0–4)	88.8	86.7		90.0	93.6		89.2	88.5	
Moderate (5–7)	9.1	11.1		8.9	6.4		9.0	9.9	
High (8–12)	2.1	2.2		1.1	0.0		1.8	1.6	

Chi-square test: n.s., not significant (*p* > 0.05); *p ≤ 0.05; **p < 0.01; ***p < 0.001. Note: The sum of numbers of men and women does not equal the total number of participants in both study samples due to missing values with respect to sex.

In the causal direction from personal life to paid work, WLC was found to be much less prevalent in both survey populations and study samples but showed the same pattern or differences between white- and blue-collar workers from different work environments except for the strain-based work-to-life conflict, with no statistically significant differences between the two survey populations (see Table 2) and study samples (see Table 3) for both sexes.

Associations between all considered WLC measures and health outcomes turned out to be fairly strong with only few exceptions, mostly graded, and statistically significant (see Table 4). The greater the exposure to such incompatibility or conflict, the higher the odds ratio or relative risk of being in poor SRH or having severe backache or sleep disorders. This was found even after adjustment for sex, age, and occupational position and basically for both study samples. However, health correlates of strain-based WLC were slightly stronger in the blue-collar worker sample, whereas health correlates of time-based WLC and working time-related incompatibility by tendency were stronger in the white-collar worker sample. This applies at least to the general and physical health outcomes used, i.e., to poor SRH and backache. As regards sleep disorders as a mental health outcome, associations and gradients were consistently stronger among low-skilled or unskilled workers. A few associations were not found to be statistically significant due to rather low numbers of cases.

**Table 4:** **Health correlates of different work-life conflict measures in white- and blue-collar workers from the service and industry sectors (study samples I and II)**.

	Poor self-rated health				Severe backache or low back pain				Severe sleep disorders			
	Study sample I		Study sample II		Study sample I		Study sample II		Study sample I		Study sample II	
	(*N* = 1,170)		(*N* = 489)		(*N* = 1,170)		(*N* = 489)		(*N* = 1,170)		(*N* = 489)	
	%	OR^a^	%	OR^a^	%	OR^a^	%	OR^a^	%	OR^a^	%	OR^a^
Total study sample	11.0		17.6		6.6		12.3		7.8		11.9	
Compatibility of work hours with private life activities		1.82***		1.53*		2.04***		1.40		2.39***		2.38***
Very good	4.9	1	13.6	1	2.5	1	11.5	1	4.0	1	7.3	1
Good	10.6	2.37*	19.3	1.50	6.5	2.85*	10.8	0.93	5.6	1.40	11.2	1.49
Not very good/ not at all good	16.7	4.08***	26.5	2.40*	10.2	4.65**	20.4	2.23	16.8	4.83***	34.7	6.28***
Time-based work-to-life conflict		1.14***		1.09*		1.15***		1.08		1.18***		1.25***
None or low (0–4)	8.3	1	14.7	1	5.3	1	10.9	1	4.8	1	7.5	1
Moderate (5–7)	13.4	1.72**	27.0	2.20**	6.6	1.30	12.5	1.19	9.9	2.23**	19.1	2.71**
High (8–12)	15.2	1.98*	20.0	1.55	11.2	2.12*	22.0	2.19	13.3	3.14***	30.0	5.13***
Strain-based work-to-life conflict		1.24***		1.30***		1.15***		1.29***		1.40***		1.45***
None or low (0–4)	5.5	1	9.0	1	4.0	1	6.7	1	2.7	1	4.9	1
Moderate (5–7)	10.9	2.12**	25.5	3.65***	7.2	1.82*	12.3	1.90	5.7	2.04*	12.3	2.59*
High (8–12)	21.2	4.59***	33.3	5.61***	10.2	2.53**	33.8	6.87***	20.2	8.73***	37.5	10.7***
Time-based life-to-work conflict		1.19**		1.13*		1.07		1.13		0.98		1.11
None or low (0–4)	9.7	1	17.3	1	6.4	1	11.0	1	7.6	1	11.0	1
Moderate (5–7)	19.1	2.26***	20.6	1.28	7.5	1.10	23.5	2.80*	9.6	1.35	17.6	1.71
High (8–12)	–	–	16.7	0.99	10.5	1.45	33.3	4.37	–	–	33.3	4.06
Strain-based life-to-work conflict		1.18***		1.49***		1.14*		1.15*		1.19***		1.27***
None or low (0–4)	9.8	1	14.7	1	6.1	1	11.7	1	6.8	1	10.2	1
Moderate (5–7)	18.4	2.11**	40.4	4.92***	8.0	1.37	12.5	1.13	17.8	3.17***	20.8	2.64*
High (8–12)	38.1	6.48***	37.5	6.51*	22.7	5.54***	50.0	6.33*	13.6	2.33	50.0	9.09**

*^a^Odds ratios adjusted for sex, age, and occupational position (job status) and calculated: (a) for each additional level or score (first number) and (b) by contrast (third and fourth number) with the reference group of the least exposed (OR = 1)*.

*p ≤ 0.05; **p < 0.01; ***p < 0.001.

In sum, although WLC was found to be much less frequent and poor health outcomes turned out to be much more prevalent among blue-collar than white-collar workers in this study, the associations or dose-response relationships were shown to be equally strong in both study samples or even stronger among blue-collar workers. The same is true for moderate or high levels of time- and strain-based life-to-work conflict, which were found to be much less prevalent (see Table 3), but in general not less strongly associated with poor health outcomes than moderate and high levels of time- and strain-based work-to life conflict, independently of the study sample (see Table 4). And although the stratified analyses did not allow to test for a statistical significance between the strata used, the calculated contrasted odds ratios nevertheless permitted conclusions to be drawn and comparisons to be made between the two study samples regarding the strength and gradient of association.

## Discussion

The present cross-sectional study aimed to make a significant contribution to the research by comparing two independent, large and heterogeneous samples of white- and blue-collar workers from different work environments with regard to diverse aspects or dimensions of WLC and their associations with general, physical and mental health outcomes. Research on work-family issues in the past has been largely limited to public servants and professional categories such as managers, freelancers, professors, teachers, and students, etc. These subjects are usually highly educated members of the middle-class who are self-employed or employed by large companies and who work in the public and/or service sector.

This study therefore contributes to the research and literature by including a formerly understudied population, namely blue-collar workers, or more precisely low-skilled and unskilled manual workers of the industrial sector and from low-income industries such as construction, machine, or metal working. Moreover, it contrasts them with white-collar employees, or more specifically with university graduates mostly in high-status occupations (e.g., private bankers, risk managers, and physicians) and working in the service sector and rather high-income industries such as banking, insurance, and healthcare.

Experiences of work-life or rather work-family conflict in low-income jobs and among blue-collar workers have been very rarely examined so far and particularly across various professional categories and different organizations and, if so, then only on the basis of very small samples from a single organization or occupation, and restricted to men or working parents (with at least one child) and/or either not in comparison with white-collar workers or not in association with health outcomes (26, 29, 30). This study, in contrast, was both comparative and health-related and was based on much larger, more heterogeneous and independent samples from several organizations and two different economic sectors including singles and childless couples.

At first, WLC in both studied forms and in the direction from personal life to paid work turned out to be much less prevalent in both survey populations and study samples than the two forms of work-to-life conflict, as has been shown and consistently found in earlier studies [see *inter alia* (28, 34)]. In addition and as expected, a considerably and significantly lower prevalence of nearly all measures and dimensions of WLC (except for strain-based life-to-work conflict) was found in the industrial work environment and, even more pronounced, among less educated industrial and construction workers (blue-collar workers) compared to the service sector and particularly to highly educated service workers (white-collar workers). This finding, although expected, contrasts with the results of a recently published study and conference paper of Francis et al. (26), who found higher mean scores of time- and strain-based work-family conflict among blue-collar than among white-collar workers in the Australian construction industry. A reason for this inconsistency may be that the white-collar workers in the Australian study were also from the construction industry, even though they were characterized as working “on site in the site office” and not “directly in a construction activity,” whereas white-collar workers in our Swiss study were defined and selected as university graduates working in the service sector and mainly in the banking or insurance industry. It is obvious that such differently characterized white-collar workers will also differ fundamentally in their working and living conditions and also in their expectations and needs regarding work-family issues. In other words: the two studies are basically not comparable with each other due to their very different study settings and samples. This applies even more to the very few other studies on work-family conflict that include or focus on blue-collar workers but did not report and compare their prevalence of work-family conflict with those of white-collar workers (27, 29), or were not quantitative but qualitative and therefore equally incomparable (30).

In general, exposure to higher levels of WLC was consistently and similarly strongly associated with considerably increased risks of poor general, physical, and mental health outcomes. This was observed basically for both study samples and by tendency across all dimensions or indicators of WLC. However, whereas the health correlates of strain-based WLC were slightly stronger among blue-collar workers, those of time-based WLC and time-related incompatibility between paid work and personal life were stronger among white-collar workers, except for severe sleep disorders, where associations and gradients were consistently stronger among blue-collar workers.

Such differentiated findings were not reported in any other study since, to my knowledge, no other comparable study has been published. And these study results suggest that lack of time or rather high time commitment to work and/or personal or family life (time-based WLC) may represent a greater health risk and be more relevant to white-collar workers, whereas exhaustion and lack of energy as a result of a physically or emotionally demanding or stressful job and/or personal life (strain-based WLC) may be more detrimental to health in blue-collar workers.

### Strengths and limitations

The study samples of white- and blue-collar workers were drawn from wider independent samples among the workforces of eight large and medium-sized companies from diverse industries and economic sectors. This, and the approach to in a way “oversample” the two distinct job types by selecting university graduates and unskilled workers from two different work environments (where such educational categories are disproportionately represented) in the first place allowed such an extreme group comparison, which was expected to reveal more pronounced results as regards possible differences between blue- and white-collar workers. Blue- and white-collar workers from the same individual companies or particular industries may work under highly similar conditions or may hardly exist in statistically sufficient quantities or proportions for both job types (manual work vs. service work). Using data from only one of the two surveys in only one economic sector would have implied a non-representative and insufficiently large subsample of either low or unskilled workers or university graduates compared to a nationally representative standard population of employees with regard to sufficient statistical power (see Table 1). In addition, such a comparison across different economic sectors and very diverse industries is more likely to provide satisfactory sampling heterogeneity and consequently study results capable of generalization.

On the other hand, one could criticize or argue that the findings are still not representative of the entire employed population or may rather reflect the differences between the two economic sectors than between the two job types. However, the findings of the present study are plausible and largely in line with theoretical considerations and research hypotheses. Moreover, it is self-evident that some industries are dominated by high-status occupations and well-paid white-collar jobs while in others low-income and blue-collar jobs predominate. In any case, such a categorization of job type (blue- vs. white-collar) or level of education (no/low vocational education vs. university degree) is like a meta- or superior variable that stands for a whole range of different job characteristics and working conditions. And the same applies to the distinction between the industrial and service sectors. This means that differences between the service and the industrial sectors or between such diverse industries as metalworking and banking may be partly or mainly explained by their very different proportions of blue- and white-collar or low and highly skilled workers (and their differing working conditions), as was observed in our two survey populations (see Table 1).

Apart from the use of nationally non-representative survey and study populations, which limit the possibility to generalize the findings, a response rate of 54% on average means that a selection bias cannot be excluded, and no causal inferences can be drawn, as with any cross-sectional study. However, the full survey of the participants in seven of the total of eight workforces and the size and heterogeneity of the two study samples as well as the strong associations and clear dose–response relationships between WLC measures and health outcomes found in both samples produce a good chance that the study findings reflect true conditions and universal differences between blue- and white-collar workers as regards the prevalence and health correlates of WLC, at least for Switzerland. There is particularly no indication of a noteworthy selection bias in the direction of self-exclusion of individuals with higher levels of WLC and/or health problems from survey participation. If, nonetheless, such a self-exclusion had occurred, this would presumably have resulted in an underestimation rather than an overestimation of the “true” prevalence of such experiences of role conflict and health problems and their association.

## Conclusion

It seems evident that negative spillover or role conflict and incompatibility between paid work and personal life is an important issue not only for white-collar workers and public servants, as commonly found in previous studies, but also for blue-collar workers, particularly if they are male. Although less prevalent, time- and strain-based WLC seems to be equally or even more detrimental to the health of blue-collar workers and may be jointly responsible for the poorer health outcomes, or rather the higher proportions of persons affected by health problems, in blue-collar compared to white-collar workers. This may have something to do with social comparisons or a relative lack of social and/or financial resources and therefore needs to be examined in more detail in future research. In any case, before it can be considered as a solid fact and certain knowledge, this finding needs to be replicated and supported by other studies, comparing not only extreme groups with regard to education but persons from all educational levels and all occupational positions.

This study has shown that low-skilled and unskilled workers from low-income industries and/or in low-status occupations also need to be taken into consideration for future research and when planning and implementing interventions, support and offers at the workplace designed to better integrate and reconcile work and personal life and hence to improve and promote the health and well-being of employees. In short, blue-collar workers need to be included in future studies and considered as a target group in worksite health promotion.

## Conflict of Interest Statement

The author declares that the research was conducted in the absence of any commercial or financial relationships that could be construed as a potential conflict of interest.
